# Prognostic Significance of Lymphovascular Invasion Detected by D2-40 in Low-Risk Stage II Colon Cancer

**DOI:** 10.7759/cureus.19825

**Published:** 2021-11-23

**Authors:** Anand Prugmahachaikul, Anapat Sanpavat

**Affiliations:** 1 Department of Pathology, Faculty of Medicine, Chulalongkorn University, Bangkok, THA

**Keywords:** immunohistochemistry, prognostic significance, d2-40, lymphovascular invasion, stage ii colon cancer

## Abstract

Background

Lymphovascular invasion (LVI) is included in the criteria of high-risk stage II colon cancer. However, there are limitations to detecting LVI by routine hematoxylin and eosin (H&E) staining. Alternatively, immunohistochemistry (IHC) for the lymphatic endothelial marker D2-40 may help detect LVI, but its prognostic significance remains unknown. This study aimed to evaluate the prognostic significance of LVI, detected by IHC for D2-40, in low-risk stage II colon cancer.

Materials and Methods

A total of 69 patients with low-risk stage II colon cancer were tested for D2-40 to assess LVI. Then, the relationships between IHC-detected LVI and clinical outcomes, including disease-free survival (DFS) and overall survival (OS), were analyzed using both univariate and multivariate analyses.

Results

IHC for D2-40 revealed that 24 out of the 69 cases (34.78%) had LVI-positive tumors. IHC-detected LVI was significantly associated with adverse clinical outcomes on univariate analysis, i.e., both reduced DFS (P = 0.002) and OS (P = 0.0163). In multivariate analysis, controlling for age, IHC-detected LVI remained a significant predictor of reduced DFS with a hazard ratio (HR) of 3.37 and a 95% confidence interval (CI) of 1.39-8.15 (P = 0.007) and OS (HR, 5.66; 95% CI, 1.02-31.51; P = 0.048).

Conclusions

Our results demonstrated that IHC analysis for D2-40 enhanced LVI detection in patients with low-risk stage II colon cancer and that cases with a missed diagnosis of LVI by routine H&E staining had adverse clinical outcomes, that is, reduced DFS and OS.

## Introduction

 Colon cancer is the third most commonly diagnosed cancer (10.2% of all cases) in both sexes combined worldwide, accounting for 1,096,601 new cases and 551,269 deaths in 2018 [1-3]. Strategies for managing the newly diagnosed cases of colon cancer depend on the pathological stage of the disease. Stage II colon cancer is any tumor extending beyond the muscularis propria into the pericolic tissue or adjacent organs without lymph node metastasis [4,5]. Basically, adjuvant chemotherapy after resection of the primary tumor should not be routinely recommended for unselected patients with stage II colon cancer. However, since clinical outcomes of high-risk stage II cases are similar to those of patients with stage III disease, high-risk stage II patients may need to receive postoperative chemotherapy with single-agent 5-fluorouracil (5-FU) [6-8]. Features of high-risk disease include inadequate lymph node sampling (< 12 nodes), poorly differentiated tumors, lymphovascular or perineural invasion, pT4 stage, and clinical presentations such as intestinal occlusion or perforation [6,8].

 Lymphovascular invasion (LVI) represents one of the criteria for a high-risk condition. LVI is an independent risk factor for unfavorable outcomes [9-11]. Currently, LVI is identified by routine hematoxylin and eosin (H&E) staining and defined as the presence of tumor emboli within thin-walled structures lined by endothelial cells without any identifiable smooth muscle layer or elastic lamina. However, it is difficult to detect LVI by using routine H&E staining. Controversies over LVI detection arise mainly from the difficulty in visualizing lymphatic vessel walls. Accordingly, immunohistochemistry (IHC), particularly for lymphatic endothelial markers such as D2-40, should increase the sensitivity of LVI detection in stage II colon cancer. D2-40 is a monoclonal antibody against a 40-kDa O-linked transmembrane sialoglycoprotein podoplanin, a specific marker for lymphatic endothelial cells [12]. Several studies have reported that D2-40 immunostaining can increase the detection rate of LVI in colon cancer [13-17]. Furthermore, previous research has revealed an association between IHC-detected LVI and clinical relevance in other tumors (e.g., breast cancer and melanoma) [18-20]. The objective of this study was to evaluate the prognostic significance of IHC-detected LVI in low-risk stage II colon cancer.

## Materials and methods

Study population

This study was performed at the Department of Pathology, Faculty of Medicine, King Chulalongkorn Memorial Hospital (Bangkok, Thailand). We used the hospital database to retrospectively review patients’ electronic medical records, surgical reports, and pathological slides. The Institutional Review Board at the Faculty of Medicine, Chulalongkorn University, approved the study protocol for using tissue and clinical data (IRB No. 805/62, January 2020). A total of 69 patients diagnosed with low-risk stage II colon cancer between 2007 and 2014 were enrolled in the study. All of them had been treated with colectomy and lymph node resection and had available paraffin blocks as well as official pathology reports. The study excluded cases with fewer than five years of follow-up. Data regarding age at initial diagnosis, gender, histological grade or subtype, date of first recurrence, and date of death were collected from electronic medical records and official surgical pathology reports.

IHC procedure

All original H&E slides were examined by two pathologists and all of the faded slides were recut to ensure the absence of LVI and the other high-risk features. To select two paraffin blocks from each patient for IHC staining, we targeted the advancing front of the tumor and the area with tumor budding. IHC analysis was performed using the commercially available lymphatic endothelial marker podoplanin (monoclonal, D2-40, Dako IR072) detected with EnVision FLEX Visualization Systems (Dako, Autostainer Link 48) (Agilent Technologies Inc., Santa Clara, California). Briefly, after deparaffinization and rehydration, the sections were pretreated with tris aminomethane-ethylenediaminetetraacetic acid (Tris-EDTA) buffer (pH 9.0) at 95°C for 20 minutes. After the sections were cooled down and immersed in Tris-EDTA buffer, they were preincubated with peroxidase blocking reagent (FLEX) for five minutes. The sections were then incubated with the ready-to-use anti-D2-40 antibody for 20 minutes, followed by incubation with horseradish peroxidase-labeled secondary antibody (FLEX). The complex was visualized with 3,3’-diaminobenzidine tetrahydrochloride solution (FLEX DAB+ chromogen), and hematoxylin was used as the nuclear counterstain. Each run included appropriate positive and negative controls.

LVI assessment

**Figure 1:**
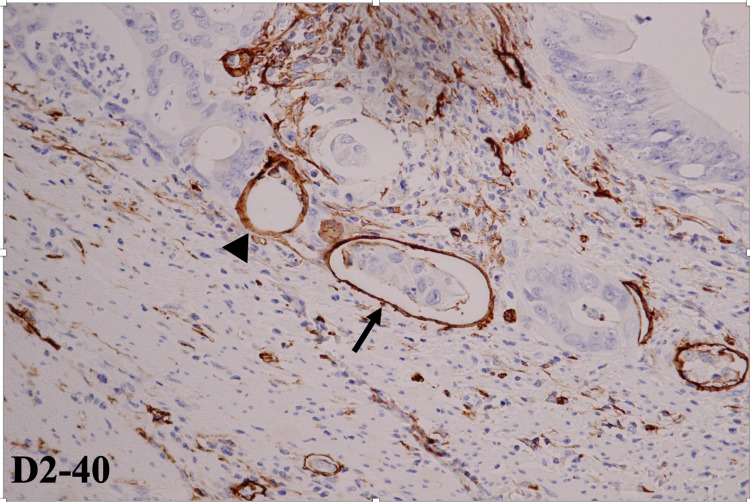
IHC-stained section using D2-40 demonstrates a lymphatic space containing a cluster of tumor cells (arrow). On the other hand, no tumor cell is present in another lymphatic space on the left side (arrowhead). Magnification: ×400. IHC: immunohistochemistry.

All D2-40-stained slides were evaluated for LVI by an experienced gastrointestinal pathologist blinded to clinical data. LVI was defined as the presence of at least one tumor cell within D2-40-stained lymphatic spaces, as shown in Figure 1. All cases were categorized as either LVI-positive or LVI-negative.

Statistical analysis

Patient characteristics, including demographic data and clinicopathological parameters, were analyzed by descriptive statistics. The two-sample t-test was used to compare mean values of numerical variables (e.g., mean age at initial diagnosis) between groups (i.e., LVI-positive and LVI-negative patients). Categorical variables, for example, frequency distributions between LVI-positive and LVI-negative cases, were evaluated using the chi-square or Fisher exact test, as appropriate.

Survival analysis was conducted to evaluate the clinical outcome of patients with low-risk stage II colon cancer. Disease-free survival (DFS) was defined as the length of time patients lived without any evidence of the disease and was calculated from the date of colon cancer diagnosis to the date of first recurrence or death. Overall survival (OS) was defined as the length of time from the date of colon cancer diagnosis until death. DFS and OS were estimated by Kaplan-Meier survival analysis, and the log-rank test was used to assess statistical significance between LVI-positive and LVI-negative cases. To evaluate the independent effect of IHC-detected LVI, we performed univariate and multivariate Cox proportional hazard regression analyses. Hazard ratios (HRs) were calculated, and 95% confidence intervals (CIs) were also presented. Proportional hazard assumptions were verified using Schoenfeld residuals and log-log plots. All P values were two-sided, with statistical significance evaluated at the 0.05 α level. All analyses were performed using Stata Statistical Software: Release 15 (2017; College Station, Texas: StataCorp LLC).

## Results

Patient characteristics

The study enrolled 69 patients with low-risk stage II colon cancer, including 37 men (53.62%). The overall median age at initial diagnosis was 66 (range 39-90) years, and nearly two-thirds of the patients (N  =  41, 59.4%) had well-differentiated tumors histologically.

Using IHC for D2-40, we identified 24 (34.78%) participants with LVI. There was no statistically significant relationship between the presence of LVI (as detected by D2-40 immunostaining) and clinicopathological variables, including age at initial diagnosis, sex, and histological grade or subtype. Table 1 summarizes the associations between LVI status and clinicopathological variables. Survival data were also recorded prospectively for all patients. Overall rates of DFS and OS were found to be 3.18 recurrences per 1,000 person-months (95% CI 2.07-4.88) and 0.91 deaths per 1,000 person-months (95% CI 0.43-1.90), respectively.

**Table 1:** Comparison of the correlation between clinicopathologic variables and LVI status in low-risk stage II colon cancer (N = 69) *By t-test, Chi-square test, or Fisher exact test, as appropriate. LVI: lymphatic vascular invasion; SD: standard deviation.

	LVI-Negative by D2-40 (N = 45)	LVI-Positive by D2-40 (N = 24)	P*
Age at Diagnosis (y)			0.90
Mean (SD)	66 (11)	66 (13)	
Gender			0.28
Male (n = 37)	22 (49)	15 (63)	
Female (n = 32)	23 (51)	9 (37)	
Histological Grade and Subtype			0.73
Well-differentiated (n = 41)	28 (62)	13 (54)	
Moderately differentiated (n = 26)	16 (36)	10 (42)	
Mucinous adenocarcinoma (n = 2)	1 (2)	1 (4)	

LVI detected using D2-40 is associated with reduced DFS and OS

**Figure 2:**
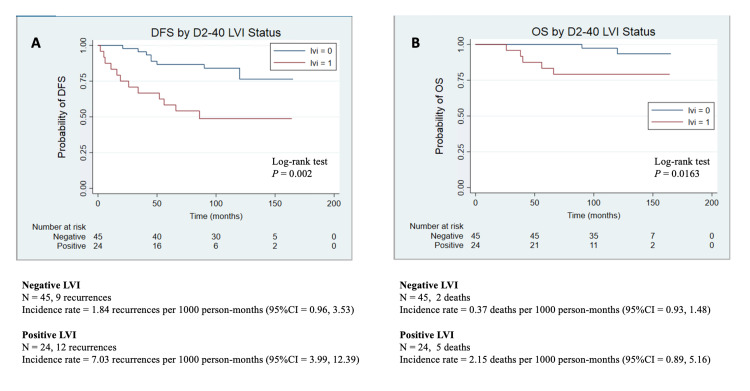
Kaplan–Meier survival curves showing (A) DFS stratified by LVI positivity as detected using D2-40 and (B) OS stratified by LVI positivity as detected using D2-40. DFS: disease-free survival; LVI: lymphatic vascular invasion; OS: overall survival; N: number

At the end of the study period, 62 (89.86%) patients were alive (48 without colon cancer and 14 with colon cancer), and there were seven (10.14%) deaths (five without colon cancer and two with colon cancer). Kaplan-Meier regression analysis and log-rank test revealed a statistically significant association between the presence of IHC-detected LVI and DFS (P = 0.002) (Figure 2A). The rate of DFS was found to be 7.03 recurrences per 1,000 person-months (95% CI 3.99-12.39) for the LVI-positive group versus 1.84 recurrences per 1,000 person-months (95% CI 0.96-3.53) for the LVI-negative group. According to Kaplan- Meier regression analysis and log-rank test results, the association between LVI and OS was also statistically significant (P  =  0.016; Figure 2B). The rate of OS was 2.15 deaths per 1,000 person-months (95% CI 0.89-5.16) for LVI-positive cases and 0.37 deaths per 1,000 person-months (95% CI 0.09-1.48) for LVI-negative cases.

In our univariate Cox proportional hazard model, age at initial diagnosis was shown to be a statistically significant factor impacting both DFS (HR 1.05; 95% CI 1.01-1.10; P = 0.021) and OS (HR 1.12; 95% CI 1.03-1.22; P = 0.008). However, in multivariate Cox regression analysis, IHC-detected LVI continued to be a significant predictor of reduced DFS (HR 3.37; 95% CI 1.39-8.15; P = 0.007) and OS (HR 5.66; 95% CI 1.02-31.51; P = 0.048). Table 2 summarizes the associations between LVI status and clinical outcomes evaluated by the age-adjusted multivariate Cox regression model.

**Table 2:** Univariate and multivariate analyses of the association between IHC-detected LVI positivity and DFS and OS LVI: lymphatic vascular invasion; IHC: immunohistochemistry; DFS: disease-free survival; OS: overall survival; HR: hazard ratio

	Univariate			Multivariate		
	HR	95% CI	P	HR	95% CI	P
DFS:						
Age	1.05	1.01–1.10	0.021	1.04	1.00–1.08	0.036
LVI-Positive by D2-40	3.63	1.51–8.68	0.004	3.37	1.39–8.15	0.007
OS:						
Age	1.12	1.03–1.22	0.008	1.10	1.024–1.19	0.010
LVI-Positive by D2-40	5.98	1.15–31.21	0.034	5.66	1.02–31.51	0.048
The associations remain statistically significant when controlling for age.						

## Discussion

The main objective of this study was to determine whether additional LVI detected by IHC staining for D2-40 significantly correlated with worse outcomes in a cohort of patients diagnosed with low-risk stage II colon cancer.

LVI detection

Our study supports the results of several previous studies reporting that IHC analysis, compared with conventional H&E staining, significantly increased the frequency of LVI detection in colon cancer. Wada et al. found that D2-40 immunostaining identified LVI in 20 out of 120 patients with T1 rectal cancer (16.67%), while H&E staining detected LVI in only seven cases [16]. Walgenbach-Bruenagel et al. showed that IHC for D2-40 increased the rates of LVI detection from 0% and 10% (using only H&E staining) to 22% and 84% in early and localized stage II and III colorectal cancer, respectively [17]. Ervine et al. reported that H&E staining alone detected lymphatic or venous invasion in six of 28 stage I colorectal cancer cases (21.4%). In contrast, double immunostaining for CD34 and D2-40 led to LVI detection in 14 patients [13]. Goodarzi et al., who examined 100 colorectal polyp cancer patients for LVI by D2-40 immunostaining and routine histology, found that using lymphatic endothelial markers increased the LVI detection rate (D2-40 immunostaining, 23% vs. routine histology, 8%). [14] In another study by Lai et al., 49 of 220 stage II colon cancer patients (22.3%) were found to have LVI by D2-40 immunostaining, whereas conventional H&E staining identified only 22 cases (10.0%) [15]. Similarly, 24 of the 69 low-risk stage II colon cancer patients (34.78%) studied herein were diagnosed as having LVI after D2-40 immunostaining, although H&E staining indicated no sign of LVI. These findings confirm that LVI sometimes fails to be detected by H&E staining alone and that IHC is an essential ancillary diagnostic tool for LVI. A summary of published articles indicating that D2-40 immunostaining aids in LVI detection is provided in Table 3.

**Table 3:** Summary of published reports demonstrating D2-40 immunostaining improved LVI detection in colorectal cancer LVI: lymphatic vascular invasion; IHC: immunohistochemistry; H&E: immunohistochemistry.

Series (Reference)	Markers	Tumor Type	No. Cases	H&E LVI (%)	IHC LVI (%)
Wada et al., 2013 [16]	D2-40	T1 colorectal cancer	120	5.83	16.67
Goodarzi et al., 2020 [14]	D2-40	Colorectal polyp cancer	100	8	23
Ervine et al., 2015 [13]	D2-40/CD34	pT1 colorectal cancer	28	21.4	50
Walgenbach et al., 2006 [17]	D2-40	Stage II colorectal cancer Stage III colorectal cancer	9 19	0 10	22 84
Lai et al., 2014 [15]	D2-40	Stage II colon cancer	220	10	22.3
Present Study	D2-40	Low-risk stage II colon cancer	69	0	34.78

Association between IHC-detected LVI and clinical outcome

Based on several studies, LVI positivity in colorectal cancer is widely accepted as an independent indicator of unfavorable prognosis [9-11]. According to the United States National Comprehensive Cancer Network, the presence of LVI is considered a high-risk feature in stage II colon cancer [6]. Furthermore, studies have demonstrated that IHC-detected LVI results in poorer clinical outcomes. In a study by Wada et al., univariate and multivariate analyses showed that LVI detection by D2-40 immunostaining was an independent risk factor for nodal metastasis in T1 colorectal cancer (OR 6.048; 95% CI 1.360-26.89; P  =  0.018) [16]. Moreover, Ishii et al. demonstrated an association between nodal metastasis and LVI positivity by IHC in T1 colorectal cancer (OR 7.12; 95% CI 2.27-22.2; P = 0.001) [21]. Lai et al. found that in stage II colon cancer, LVI positivity by D2-40 immunostaining was associated with a significantly increased risk of death (HR 2.457; 95% CI 2.032-4.652; P = 0.008), as compared to LVI positivity by H&E staining (HR 1.543; 95% CI, 0.876-1.992; P = 0.961). [15] Goodarzi et al. investigated the presence of LVI by D2-40 immunostaining in 100 patients with colorectal polyp cancer and discovered that IHC-detected LVI was associated with worse disease-specific survival (HR, 14.07; 95% CI 1.57-125.97; P = 0.018) [14]. In our study, multivariate analysis demonstrated that a missed diagnosis of LVI by routine H&E staining in stage II colon cancer was significantly associated with adverse clinical outcomes, that is to say, reduced OS and DFS. Table 4 summarizes published studies supporting that LVI positivity by D2-40 immunostaining is correlated with adverse clinical outcomes. Therefore, this study aims to determine whether IHC for anti-D2-40 monoclonal antibody should be routinely performed in low-risk stage II colon cancer patients when LVI could not be identified by routine H&E staining and whether adjuvant therapy would improve the clinical outcomes of low-risk stage II colon cancer cases with LVI positivity by D2-40 immunostaining.

**Table 4:** Summary of published reports demonstrating LVI IHC-detected LVI was associated with worse clinical outcomes in colorectal cancer LVI: lymphatic vascular invasion; IHC: immunohistochemistry; HR: hazard ratio; DFS: disease-free survival; OS: overall survival.

Series (Reference)	Markers	Tumor Type	Clinical Outcome
Wada et al., 2013 [16]	D2-40	T1 colorectal cancer	Nodal metastasis (OR 6.048; 95% CI 1.360–26.89; P = 0.018)
Ishii et al., 2009 [21]	D2-40	T1 colorectal cancer	Nodal metastasis (OR 7.12; 95% CI 2.27–22.2; P = 0.001)
Goodarzi et al., 2020 [14]	D2-40	Colorectal polyp cancer	Worse disease-specific survival (HR 14.07; 95% CI 1.57–125.97; P = 0.018)
Lai et al., 2014 [15]	D2-40	Stage II colon cancer	Increased risk of death (HR 2.457; 95% CI 2.032–4.652; P = 0.008)
Present Study	D2-40	Low-risk stage II colon cancer	Reduced DFS (HR 3.37; 95% CI 1.39–8.15; P = 0.007) and OS (HR 5.66; 95% CI 1.02–31.51; P = 0.048)

## Conclusions

The major limitations of our study were its single-institution, retrospective design, and a quite small number of participants. Therefore, the risk of false-positive results, or type I errors, may be concerned. Nonetheless, our results are in agreement with several related studies. In summary, IHC staining for the lymphatic endothelial marker D2-40 significantly enhanced LVI detection in patients with low-risk stage II colon cancer, and LVI-positive cases failing to be detected by routine histology had significantly worse clinical outcomes (i.e., reduced DFS and OS). Thus we propose the use of D2-40 immunostaining for the evaluation of LVI in every LVI-negative case by focusing on the advancing front or the tumor budding.
